# Synthesis of active cytokinins mediated by LONELY GUY is associated with cell production during early fruit growth in peach [*Prunus persica* (L.) Batsch]

**DOI:** 10.3389/fpls.2023.1155755

**Published:** 2023-04-20

**Authors:** Mary Sutton, Bayleigh Roussel, Dario J. Chavez, Anish Malladi

**Affiliations:** ^1^Department of Horticulture, University of Georgia, Athens, GA, United States; ^2^Department of Horticulture, University of Georgia, Griffin, GA, United States

**Keywords:** cytology, fruit development, fruit size, gene expression, phytohormone

## Abstract

Early fruit growth in peach is characterized by cell production. Cytokinins have established roles in regulating cell division and may regulate cell production during early fruit growth. However, the role of active cytokinins and regulation of their metabolism are not well characterized in the peach fruit. In this study, fruit growth parameters, concentrations of active cytokinin bases and a cytokinin riboside, and expression of three key cytokinin metabolism-related gene families were determined during early fruit growth. Early fruit growth was associated with intensive cell production until around 40 days after full bloom. During the early stages of this period, *trans*-zeatin (*t*Z), isopentenyladenine (iP), dihydrozeatin (DHZ) and *t*Z-riboside (*t*ZR), displayed higher abundance which declined rapidly by 3.5- to 16-fold during the later stages. Changes in concentration of active cytokinin bases were consistent with roles for them in regulating cell production. Expression analyses of members of cytokinin biosynthesis-related gene families, *ISOPENTENYL TRANSFERASE* (*IPT*) and *LONELY GUY* (*LOG*), further indicated that mechanisms of synthesis of cytokinin metabolites and their activation are functional within the fruit pericarp. Changes in expression of multiple members of the *LOG* family paralleled changes in active cytokinin concentrations. Specifically, transcript abundance of *LOG3* and *LOG8* were correlated with concentrations of *t*Z, and iP and DHZ, respectively, suggesting that the direct activation pathway is an important route for active cytokinin base synthesis during early fruit development. Transcript abundance of two *CYTOKININ OXIDASE* (*CKX*) genes, *CKX1* and *CKX2*, was consistent with roles in cytokinin catabolism during later stages of early fruit growth. Together, these data support a role for active cytokinins synthesized in the fruit pericarp in regulating early fruit growth in peach.

## Introduction

Fruit growth in peach is divided into three main stages (Connors, 1919; Crane, 1969; DeJong and Goudriaan, 1989). Fruit growth immediately after seed set is largely achieved through cell production (cell division across a population) and partially through cell expansion, and this phase is referred to as Stage I. This is followed by a lag phase during which pit lignification, and endosperm and embryo growth occur (Stage II). After the lag phase, a second period of rapid growth mediated largely by cell expansion contributes substantially to increase in fruit size (Stage III) (Connors, 1919; Day and DeJong, 1998; Lockwood and Coston, 2005). The number of cell divisions occurring within the pericarp during Stage I growth contributes greatly to the final size of the fruit (Scorza et al., 1991; Grossman and DeJong, 1995; Wu et al., 2005). Hence, factors regulating the rate and extent of cell production during this early fruit development period can ultimately influence final size of the peach fruit.

Cytokinins are a large class of phytohormones that play a major role in regulating cell division (Kieber and Schaller, 2014). Cytokinin metabolite concentration is often higher in actively proliferating tissues. In tomato (*Solanum lycopersicum* L.), the concentration of several active cytokinins is greater during the early stages of fruit development (Matsuo et al., 2012). In kiwifruit (*Actinidia deliciosa*), concentration of active cytokinins is greater in pollinated fruit that display greater cell division, than in unpollinated fruit (Lewis et al., 1996). Cytokinin deficiency in Arabidopsis [*Arabidopsis thaliana* (L.) Heynh.] through enhanced expression of cytokinin degradation enzymes results in reduced shoot growth and decreased leaf size (Werner et al., 2003). Exogenous applications of synthetic cytokinin analogues affect fruit growth in various plant species. In apple (*Malus × domestica* Borkh.), application of 6-benzyl adenine (6-BA) increases fruit size through direct stimulation of cell production in the fruit cortex (Wismer et al., 1995). In tomato, application of CPPU [N-(2-chloro-4-pyridyl)-N´-phenylurea] induces parthenocarpic fruit development through the stimulation of cell division (Matsuo et al., 2012). In kiwifruit (*Actinidia chinensis* Planch.) and pear (*Pyrus communis* L.), post-bloom applications of CPPU result in increased fruit growth (Iwahori et al., 1988; Patterson et al., 1993; Flaishman et al., 2001). Together, these studies demonstrate that endogenous and exogenously applied cytokinins influence cell division in plants and can specifically contribute to regulating early fruit growth.

Natural cytokinins are adenine derivatives with side chains attached at the *N^6^* position of the purine ring. They exist as free bases, their corresponding nucleotides and nucleosides, and as conjugated forms, together constituting the cytokinin metabolite pool (Sakakibara, 2006; Kieber and Schaller, 2014). Steady-state concentrations of cytokinins in a tissue are determined by the coordinated action of its metabolic pathways (biosynthesis, degradation and conjugation) and transport. Multiple enzymes contribute to cytokinin metabolism in plants, with isopentenyltransferases (IPTs) catalyzing the first and rate limiting step of their biosynthesis. IPTs use DMAPP (dimethylallyl diphosphate) and ADP/ATP (adenosine di/triphosphate) as substrates in higher plants to generate isopentenyladenosine-5′-diphosphate (iPRDP), and isopentenyladenosine-5′-triphosphate (iPRTP), respectively (Hwang and Sakakibara, 2006; Kieber and Schaller, 2014). Increased *IPT* expression can result in greater cytokinin synthesis. For example, expression of an *IPT* gene from *Agrobacterium tumefaciens* in tomato increased overall cytokinin concentrations and improved fruit set (Martineau et al., 1994; Martineau et al., 1995). The iP nucleotides can be converted into trans-zeatin (*t*Z) nucleotides by the action of a class of cytochrome P540 enzymes (CYP735A). Cytokinin nucleoside monophosphates (such as iPRMP and *t*ZRMP) are converted to their respective active bases either through a two-step process mediated by a nucleotidase and a nucleosidase, or through a direct activation pathway (Hwang and Sakakibara, 2006; Kieber and Schaller, 2014). The direct activation pathway involves a single step where the cytokinin nucleoside monophosphate precursor is converted to the active cytokinin base through phosphoribohydrolase activity (Kurakawa et al., 2007). In Arabidopsis and rice (*Oryza sativa*), the direct activation pathway functions as a primary mechanism of conversion of cytokinin nucleotides to free active bases (Kurakawa et al., 2007; Kuroha et al., 2009; Tokunaga et al., 2012). The direct activation pathway is mediated by members of the LONELY GUY (LOG) family of proteins first identified in rice in relation to shoot apical meristem development (Kurakawa et al., 2007). Overexpression of *LOG* increases cell division during early growth in Arabidopsis, particularly in the embryo (Kuroha et al., 2009). The *t*Z cytokinins, *t*ZRMP, *t*ZR and *t*Z, can be converted into dihydrozeatin riboside monophosphate (DHZMP), DHZR (riboside) and DHZ (free base), respectively, through the action of zeatin reductase (Sakakibara, 2006). Cytokinin biosynthesis, though initially thought to occur primarily in roots, is now known to occur in multiple organs and tissues within the plant (Miyawaki et al., 2004). Down-regulation of cytokinin abundance can be achieved through its catabolism or *via* its conjugation to sugars (glucose – Glc). Cytokinin oxidase/dehydrogenase (CKX) is a critical enzyme involved in irreversible cytokinin degradation through cleavage of the *N^6^* side chain of cytokinin bases, such as that of *t*Z, yielding adenine and 3-methyl-2-butenal (Schmülling et al., 2003; Avalbaev et al., 2012). *CKX* is often highly expressed in regions undergoing active cell division indicating that they can aid in fine-tuning tissue cytokinin concentrations (Avalbaev et al., 2012).

Owing to their known roles in regulating cell division, concentrations of active cytokinins (the free bases; Lomin et al., 2015; Romanov and Schmülling, 2022) are expected to be specifically high during stages of rapid cell division-mediated fruit growth. Further, it is hypothesized that specific members of gene families associated with cytokinin metabolism contribute to changes in active cytokinin concentration during the early fruit development period. However, only a few studies have described the abundance of cytokinins during early stages of fruit growth and development (Lewis et al., 1996; Matsuo et al., 2012). A previous study in peach described the concentrations of multiple cytokinin metabolites during early fruit growth (Arnau et al., 1999). However, cytokinins identified as the major metabolites in that study are ones with very low or no known cytokinin activity. Additionally, expression patterns of key cytokinin metabolism-related genes during early peach fruit growth have not been previously characterized, even though members of certain gene families have been identified (Immanen et al., 2013). Hence, the major goal of this study was to investigate changes in the concentrations of active cytokinins, and that of a primary transport form, *t*ZR, during early peach fruit growth. Additionally, the relationship between active cytokinin concentration and cell production, and the expression of three key cytokinin metabolism-related gene family members in regulating cytokinin concentration during early fruit development were investigated.

## Materials and methods

### Plant material

Twelve 10-year-old ‘Cresthaven’ trees at Lane’s Southern Orchard in Fort Valley, GA, were used for this study in 2019 (*n* = 4). Care and maintenance of the trees was performed according to standard commercial cultivation practices for GA by the orchard crew (Blaauw et al., 2022). The 12 ‘Cresthaven’ trees were split into blocks of three trees (four blocks). Within each block, trees were randomly assigned to one of three treatments: thinning at 22 days after full bloom (DAFB), thinning at 29 DAFB, and no thinning (control). All thinning was performed manually at the indicated dates to 15 cm spacing between fruit on a shoot. Fruit growth and temporal gene expression data presented here for ‘Cresthaven’ are from the control treatment. In 2021, seven-year-old ‘O’Henry’ trees at the Horticulture Research Farm, University of Georgia in Watkinsville, GA were used (*n* =3; four trees were used as one experimental unit). Manual thinning was not performed on ‘O’Henry’ trees used in 2021.

### Sample collection

In 2019, ten flowers were randomly tagged on each ‘Cresthaven’ tree at bloom. At each sampling date, fruit diameter was measured at two locations: cheek to cheek, and in a perpendicular orientation. If a tagged fruit was missing at the sample date (due to fruit drop), the next nearest fruit was tagged in its place. At each sample date, 3–10 blooms/fruit (based on the developmental stage) were collected at random and transported to the laboratory (Athens, GA) to obtain individual fruit weight. At each sample date, 3–10 fruit were collected at random, immediately frozen in liquid N_2_ and stored at −80°C until further analysis (*n* = 4; gene expression analyses). Samples were collected at 0, 6, 12, 19, 26, 52, 95, 125, 135 DAFB with the last two dates being harvest dates. Fruit samples from the latter three sample dates were not processed further as the focus of this study was on early fruit development. Data for the sampling dates, 6, 12, 19, 26 and 52 DAFB are presented here as limited tissue availability resulted in low quality total RNA from samples at 0 DAFB. In 2021, four ‘O’Henry’ trees were used as one experimental unit (*n* = 3). Eight fruit from each replicate were harvested randomly at each date used in this study (10, 17, 40, and 59 DAFB), and transported to the laboratory. Fruit were sliced vertically to obtain a longitudinal profile of the fruit, and scanned on a flatbed scanner (Epson V600, Epson America Inc., Los Alamitos, CA) at a resolution of 600 pixels per inch. Scanned images were analyzed using ImageJ software (NIH, USA) as described below. Fruit tissue was frozen in liquid N_2_ and stored at −80°C until further analyses (cytokinin quantification and gene expression analyses). Three fruit from each replicate were stored in CRAF III fixative (0.3% chromic acid, 2% acetic acid and 10% formalin) and used for cytology (cell number and size measurements).

### Cytology and image analyses

Fruit (‘O’Henry’) were sectioned using a vibrotome (7000smz-2 Vibrotome, Campden Instruments, Loughborough, UK) at sampling date-specific thickness ranging from 12 µm to around 50 µm. Sections were stained either in methylene blue (0.1%, aqueous) or with Calcofluor White (1 g L^−1^ with Evans Blue 0.5 g L^−1^). Sections were mounted and visualized using an Olympus BX 51 microscope. A DP70 camera was used for brightfield microscopy (methylene blue stained sections) while a RisingCam fluorescence camera was used for fluorescence imaging (Calcofluor White stained sections).

Images of scanned fruit and those obtained through microscopy analyses were analyzed using ImageJ. The fruit outline was selected manually and measured to obtain the fruit area (longitudinal sectional area). Pericarp area was determined by selecting the outline of the seed locule, measuring it and then subtracting it from total fruit area. To determine cell area, outlines of cells were manually drawn using ImageJ and the average cell area was determined. Cell area was determined at three locations within the fruit flesh: outer, mid- and inner regions. From each region at least 100 cells were used for cell area measurements. The average of these measurements is presented here. Cell number was determined by dividing the pericarp area by average cell area.

### Cytokinin quantification

Cytokinin quantification (‘O’Henry’ fruit) was performed at the BioAnalytical Facility, Bio-Discovery Unit, University of North Texas. Phytohormone extraction was performed based on Šimura et al. (2018). Quantification of specific cytokinins was performed using ultra high-performance liquid chromatography (UHPLC; Agilent 1290 Infinity II, Agilent, Santa Clara, CA), tandem mass spectrometry (MS/MS; ABSciex QTRAP6500+ triple quadrupole). Compound separation was achieved using an AQUITY UPLC CSH C18 RP column (150 mm × 2.1 mm). The mobile phase consisted of binary gradients of acetonitrile with 0.01% formic acid (A) and aqueous formic acid (0.01%; B). The flow rate was maintained at 0.5 mL min^−1^. Internal standards used were: [^15^N_4_] *trans*-zeatin and [^3^H_5_] – indole acetic acid. An external standard mix prepared using known concentrations of the targeted metabolites was used for quantification of the cytokinin metabolites.

### Gene selection and primer design

Three gene families associated with cytokinin metabolism were selected for this study: *IPT, LOG*, and *CKX.* Gene sequences in peach were identified by searching the Genome Database for Rosaceae (GDR: https://www.rosaceae.org/) using corresponding protein sequences from *Arabidopsis* (TAIR: arabidopsis.org). Selected genes were numbered based on their order in the genome. Information on gene identity, accession numbers, and primer sequences are presented in **Table S1**. Sequence information obtained through GDR was used to design primers for quantitative RT-PCR analyses.

### RNA extraction and quantitative RT-PCR

RNA extraction was performed following the procedure described by Vashisth et al. (2011). Extractions were performed on tissue samples collected at 0, 6, 12, 19, 26, and 52 DAFB in 2019 (‘Cresthaven’) and at 10, 17, 40 and 59 DAFB in 2021 (‘O’Henry’). RNA quantity and quality were determined using a NanoDrop 8000 spectrophotometer and gel electrophoresis. cDNA was prepared using ImpromII reverse transcriptase (Promega, Madison, WI). Quantitative RT-PCR analyses were performed on Stratagene Mx3005P and Aria Mx (Agilent, Santa Clara, USA). Melt curve analyses were performed to determine primer specificity. The *β-ACTIN*, *Ky-ACTIN*, and *RNA POLYMERASE II* genes were used as reference genes for ‘Cresthaven’ samples (2019), while *β-ACTIN* and *RNA POLYMERASE II* were used as reference genes for ‘O’Henry’ (2021) samples (**Table S2**; Wang et al., 2016; Zhang et al., 2016; Haider et al., 2018). Relative quantity (RQ) and normalized RQ (NRQ) values were obtained as described previously (Jing and Malladi, 2020).

### Statistical analyses

All statistical analyses were performed in JMP (SAS Institute Inc., Cary, NC). Analysis of variance (ANOVA) followed by Tukey’s HSD were used to determine significant differences across fruit development. Cytokinin quantification data and fruit growth parameters data were log transformed prior to analyses. Gene expression data (NRQ) were log_2_ transformed prior to analyses. Collection dates were analyzed separately when determining treatment effects (thinning treatments in ‘Cresthaven’). Correlation analyses among cytokinin metabolites (log transformed), and between gene expression (log_2_ transformed NRQ values) and cytokinin metabolites were performed using JMP. Owing to multiple correlations analyzed (74), adjustment for false discovery rate (FDR) was performed using the Benjamini-Hochberg method and the adjusted *P* values were used to determine significant correlations (FDR < 0.05).

## Results

### Fruit growth, cell production, and cell expansion

**Figure 1:**
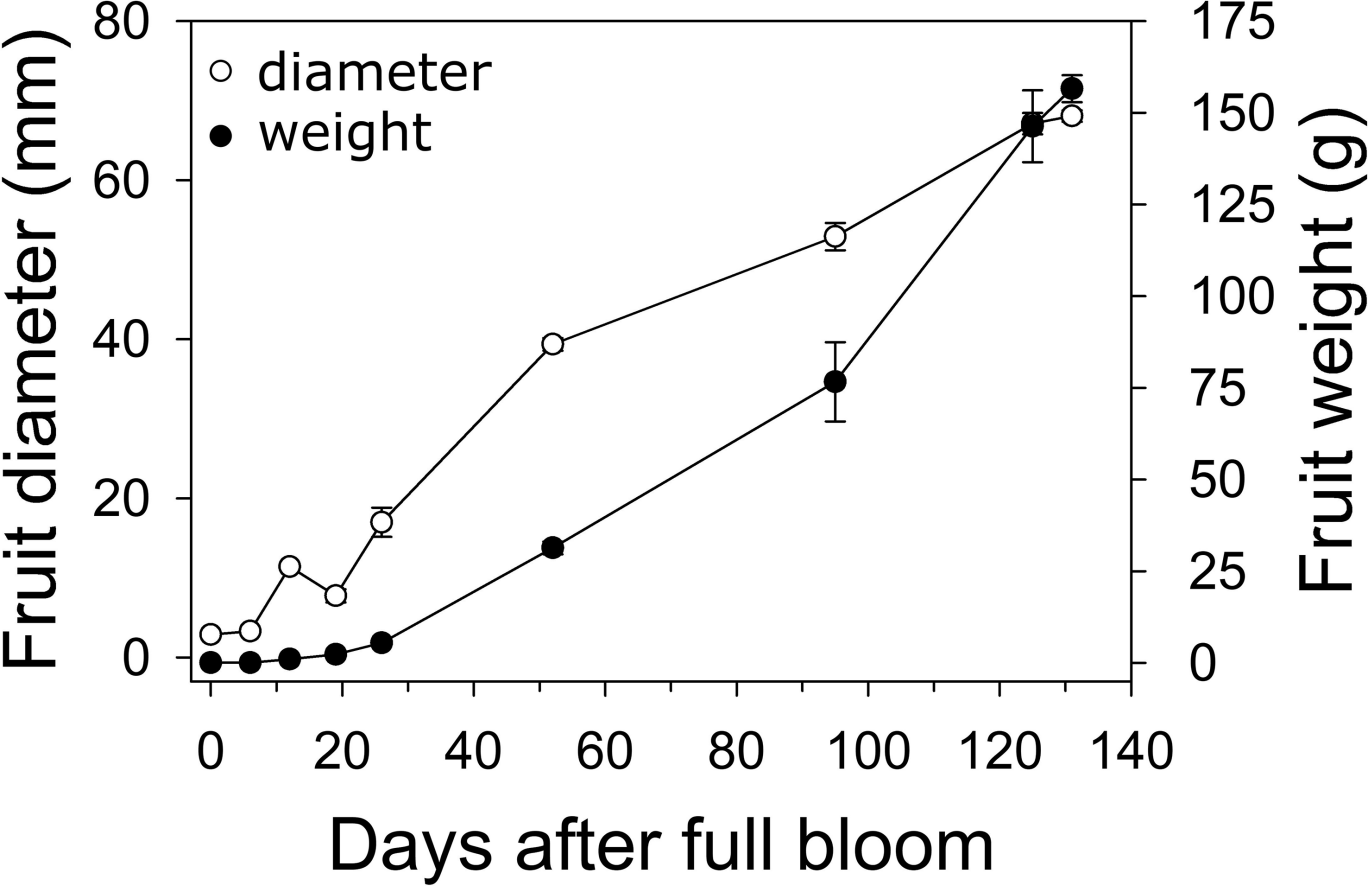
Fruit growth in ‘Cresthaven’ peach. Fruit diameter and weight were measured in ‘Cresthaven’ fruit at various stages of fruit development (*n* = 4).

In 2019, fruit diameter in ‘Cresthaven’ fruit increased by 2.7-fold between bloom and 19 DAFB, by around 5-fold between 19 and 52 DAFB, and by 1.7-fold between 52 DAFB and harvest (**Figure 1**). Over the corresponding durations, fruit weight increased by 52.9, 13.5 and 4.7-fold, respectively. Fruit relative growth rate (RGR – fresh weight basis) was highest at 12 DAFB and declined rapidly thereafter.

**Figure 2:**
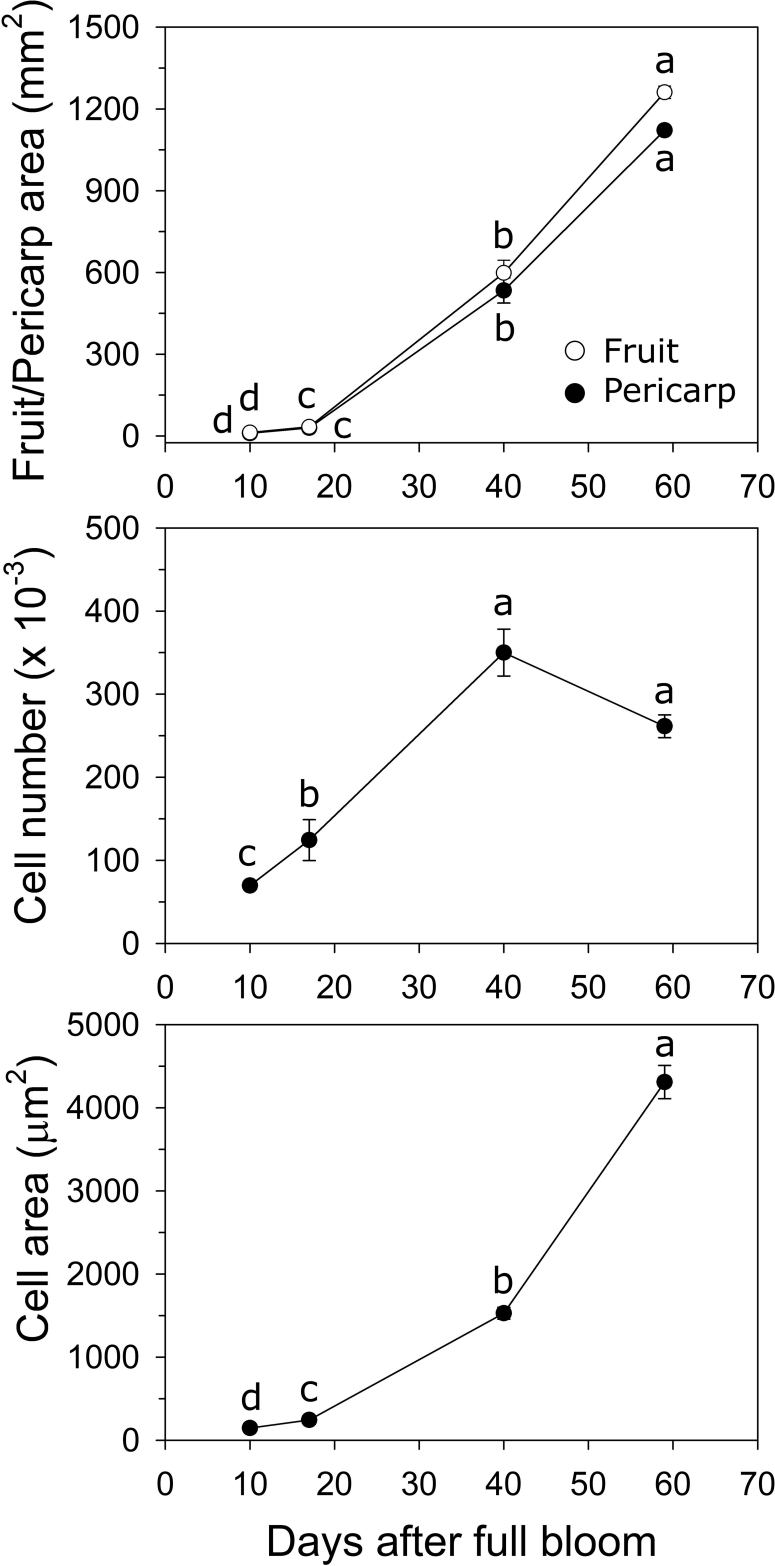
Fruit growth in ‘O’Henry’ peach. Fruit and pericarp sectional area (longitudinal) were determined using ImageJ at various stages of early fruit development. Cell number and size were determined using cytology and ImageJ analyses (*n* =3). Data were analyzed using ANOVA followed by Tukey’s HSD. Similar letters above or next to the symbols indicate that the stages are not significantly different.

In ‘O’Henry’, fruit and pericarp growth were determined primarily during early fruit development. Fruit cross-sectional area and pericarp cross-sectional area displayed very similar patterns of growth (**Figure 2**). They increased by 2.9- and 2.8-fold between 10 and 17 DAFB, by 18-fold each between 17 and 40 DAFB, and by 2.1-fold each between 40 and 59 DAFB. Fruit and pericarp RGR (area) was high during early fruit development and declined sharply by 59 DAFB. Estimated cell number across the pericarp cross-sectional area increased steadily between 10 and 40 DAFB and was subsequently not significantly altered (**Figure 2**). The highest relative cell production rate (RCPR) was noted at 17 DAFB (0.078 d^−1^) and declined by the late stages studied (59 DAFB). Cell size increased over the entire duration of the study, particularly between 17 and 40 DAFB by 6.3-fold (**Figure 2**). The relative cell expansion rate (RCER) was high during early stages of fruit development, specifically between 17 and 40 DAFB (0.08 d^−1^) but was not significantly different across the fruit developmental stages studied.

### Cytokinin metabolite concentration during early fruit development

**Figure 3:**
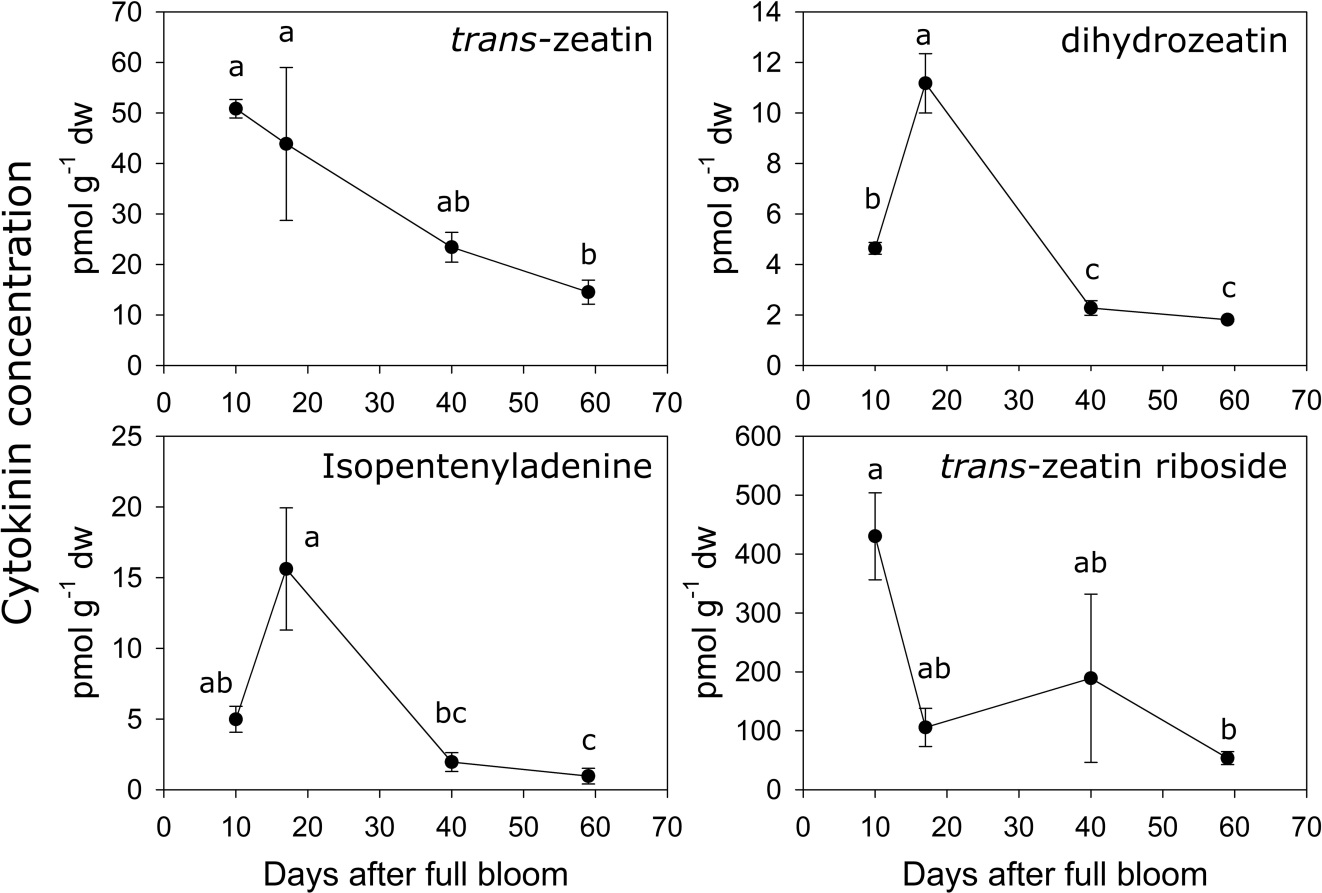
Quantification of cytokinin-related metabolites. Concentration (pmol g^−1^ dry weight) of various cytokinin-related metabolites were determined using UPLC-MS/MS in ‘O’Henry’ peach fruit during early stages of fruit development. Mean and standard error (*n* = 3) of metabolites are presented. The four time points of fruit development were analyzed using analysis of variance (ANOVA) followed by Tukey’s HSD. Similar letters above symbols indicate that corresponding stages are not significantly different.

The concentrations of several metabolites within the cytokinin biosynthesis pathway were quantified during early fruit development in ‘O’Henry’ fruit. In general, concentrations of the cytokinin metabolites were highest during early fruit development and declined during later fruit development (**Figure 3**). Among the active cytokinins (nucleobases), *trans*-zeatin (*t*Z) displayed the highest concentration during early fruit development, while isopentenyladenine (iP) and dihydrozeatin (DHZ) displayed relatively lower concentrations. The concentration of *t*Z was relatively higher during the early stages studied here (10–17 DAFB) and decreased by over 3-fold by 59 DAFB. DHZ and iP displayed similar profiles, reaching maximum concentrations at 17 DAFB and subsequently declining greatly between 17 and 40 DAFB (around 5- and 8-fold decline, respectively). Concentration of the nucleoside, *t*ZR, was also determined as it was reported as one of the major ribosides in peach fruit and is a key form of cytokinin transport (Arnau et al., 1999; Osugi et al., 2017). Concentration of *t*ZR was high during early fruit development (highest at 10 DAFB) and substantially lower at 59 DAFB, declining by around 8-fold during this period. *t*ZR abundance was up to 8-fold higher than that of *t*Z during early fruit development.

Analyses of correlations among the cytokinin metabolites indicated that *t*Z concentration was correlated with that of iP, DHZ and *t*ZR (Spearman’s *ρ* of 0.76, 0.75 and 0.78, respectively). Further, iP and DHZ concentrations were strongly correlated (*ρ =* 0.97), but neither was correlated with that of *t*ZR.

### Transcript abundance of *IPT* genes during early fruit development

**Figure 4:**
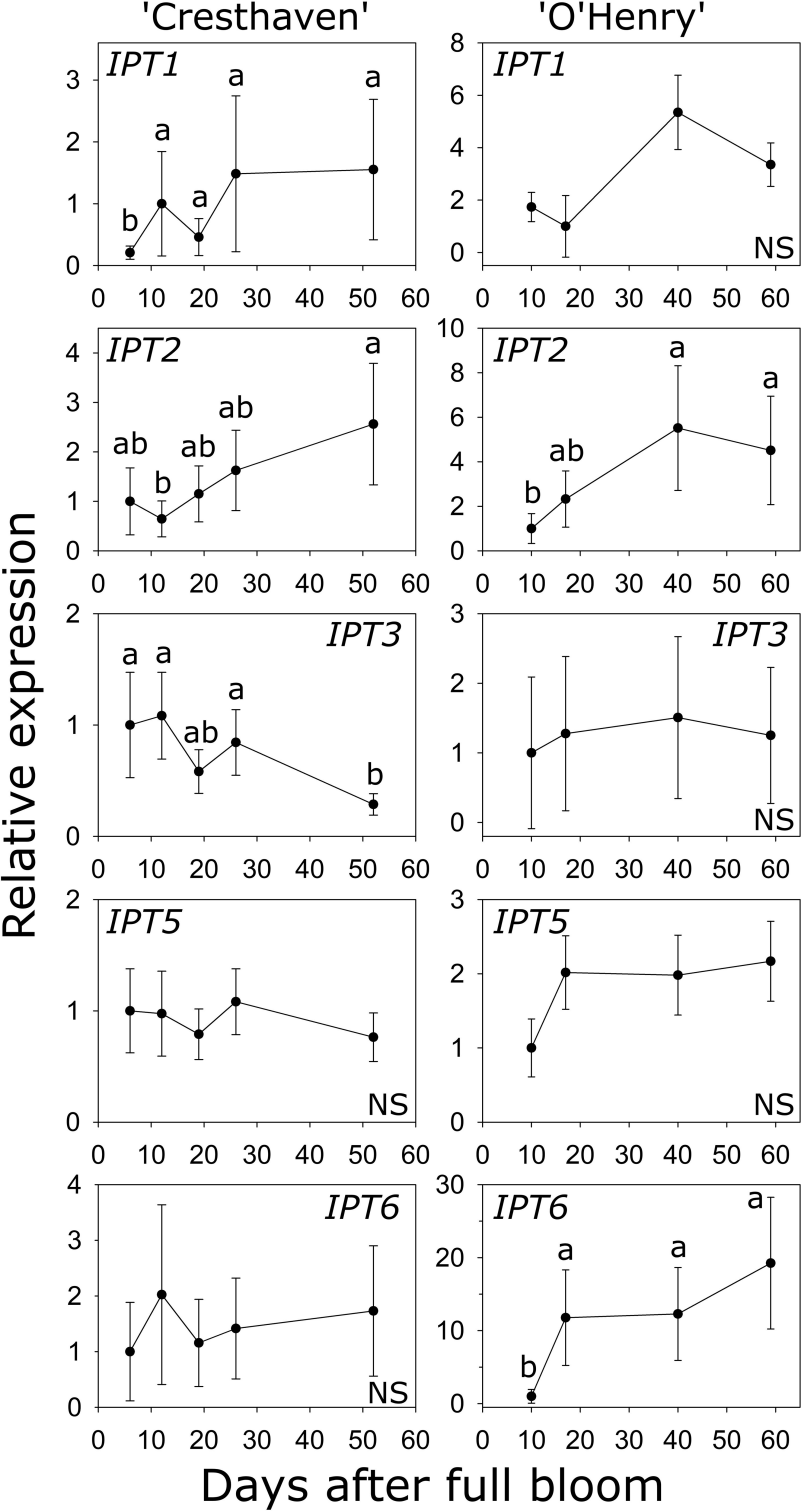
Transcript abundance of *ISOPENTENYL TRANSFERASE (IPT)* genes in two peach genotypes. Transcript abundance was determined using quantitative RT-PCR with *β-ACTIN*, *KyACTIN*, and *RNA POLYMERASE II* as reference genes for ‘Cresthaven’ (*n* = 4), and *β-ACTIN* and *RNA POLYMERASE II* for ‘O’Henry’ (*n* = 3). Transcript abundance is displayed relative to that at the first stage of fruit development used. Stages of fruit development were compared using ANOVA followed by Tukey’s HSD. Similar letters above the symbols indicate that the stages are not significantly different. NS: not significantly different across stages.

Six *IPT* genes were identified in peach and transcript abundance of five of these genes could be studied during early fruit development (**Table S1**; **Figure 4**). Transcript abundance of *IPT1* was lowest at 6 DAFB (2.2- to 7.4-fold lower than at later stages) and increased later in ‘Cresthaven’ but was not significantly altered in ‘O’Henry’. Abundance of *IPT2* transcripts was low at 12 DAFB compared to that at 52 DAFB (4-fold) in ‘Cresthaven’, and lower at 10 DAFB (up to 2.8-fold) compared to that at 40 and 59 DAFB in ‘O’Henry’. Abundance of *IPT3* transcripts was high during most of early fruit development but was reduced by up to 3.8-fold at 52 DAFB in ‘Cresthaven’. It was not significantly altered in ‘O’Henry’. *IPT5* transcript abundance was not significantly altered during fruit development in both cultivars. *IPT6* transcript abundance was not significantly altered in ‘Cresthaven’. In ‘O’Henry’, its abundance increased by over 11-fold between 10 and 17 DAFB and subsequently remained high. *IPT6* transcript abundance was negatively correlated with *t*Z concentration (*ρ =* −0.74).

### Transcript abundance of *LOG* genes during early fruit development

**Figure 5:**
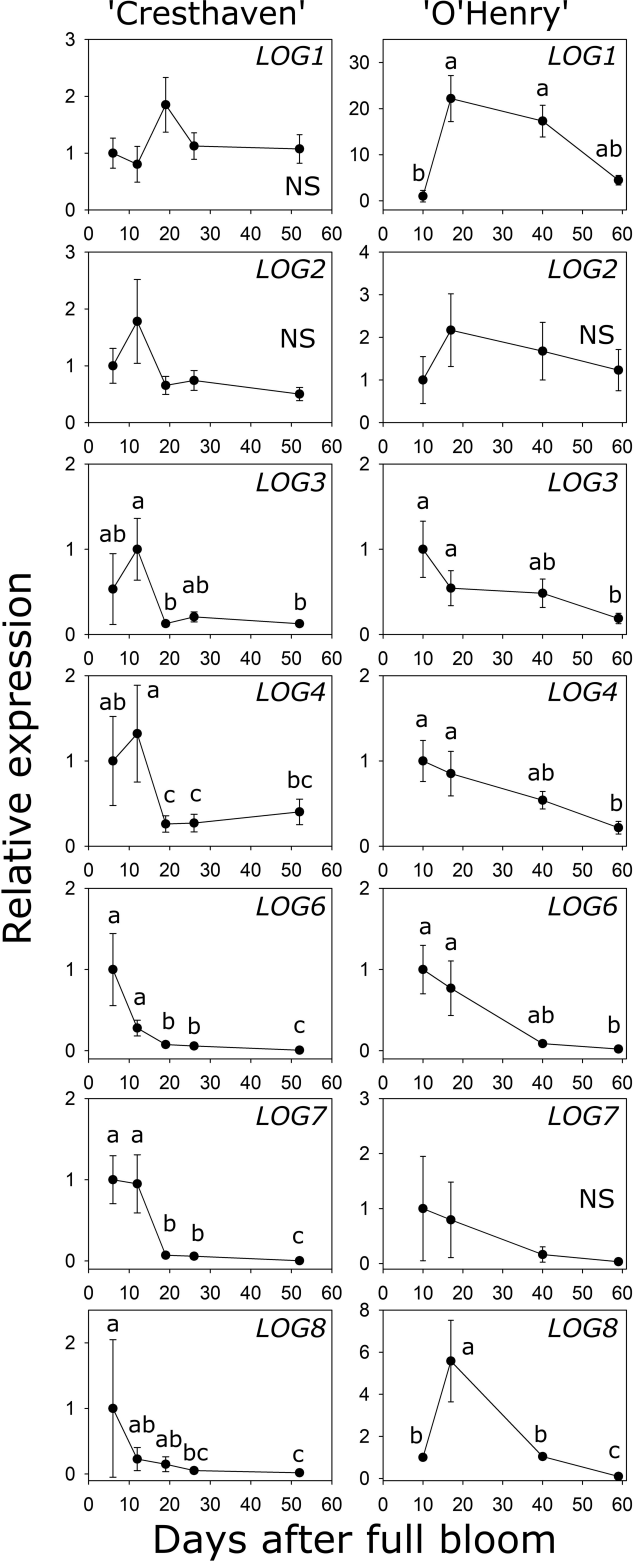
Transcript abundance of seven *LONELY GUY (LOG)* genes in two peach genotypes. Transcript abundance was determined using quantitative RT-PCR. *β-ACTIN*, *KyACTIN*, and *RNA POLYMERASE II* were used as reference genes for ‘Cresthaven’ (*n* = 4), and *β-ACTIN* and *RNA POLYMERASE II* were used for ‘O’Henry’ (*n* = 3). Transcript abundance is presented relative to that at the first stage of fruit development. Stages of fruit development were analyzed using ANOVA followed by Tukey’s HSD. Similar letters above the symbols indicate that the stages are not significantly different. NS: not significantly different across stages.

Eight *LOG* genes were identified in peach and the transcript abundance of seven of them could be investigated during fruit development (**Table S1**; **Figure 5**). *LOG1* transcript abundance was not significantly altered in ‘Cresthaven’ but increased by over 22-fold between 10 and 17 DAFB in ‘O’Henry’. *LOG2* abundance did not significantly change in either cultivar. In ‘Cresthaven’, *LOG3* transcript abundance was high during early fruit development (12 DAFB) and declined by 7.8-fold by 19 DAFB and remained low thereafter. In ‘O’Henry’, its abundance was higher during early fruit development and declined by over 5-fold by 59 DAFB. In ‘Cresthaven’ abundance of *LOG4*, *LOG6* and *LOG7* transcripts was high during early fruit development, and declined by 5-, 3.7- and 13.5-fold, respectively between 12 and 19 DAFB. Thereafter, they remained low or decreased further. In ‘O’Henry’, *LOG4* and *LOG6* abundance was high early during fruit development and reduced by up to 4.6- and 50-fold by 59 DAFB. Abundance of *LOG7* was not altered significantly during ‘O’Henry’ fruit development. In ‘Cresthaven’, transcript abundance of *LOG8* gradually decreased during fruit development by over 54-fold. In ‘O’Henry’, its abundance initially increased between 10 and 17 DAFB by 5.6-fold and then declined gradually over fruit development by over 58-fold.

Transcript abundance of *LOG3* was correlated with the concentration of *t*Z (*ρ* = 0.71). *LOG4* transcript abundance was correlated with the concentration of *t*Z and DHZ (*ρ* = 0.82 and 0.70, respectively), while that of *LOG6* was correlated with the concentration of *t*Z, DHZ and iP (*ρ* = 0.80, 0.85 and 0.80, respectively). Further, abundance of *LOG8* was correlated with the concentration of DHZ and iP (*ρ* = 0.89 and 0.90, respectively).

### Transcript abundance of *CKX* genes during early fruit development

**Figure 6:**
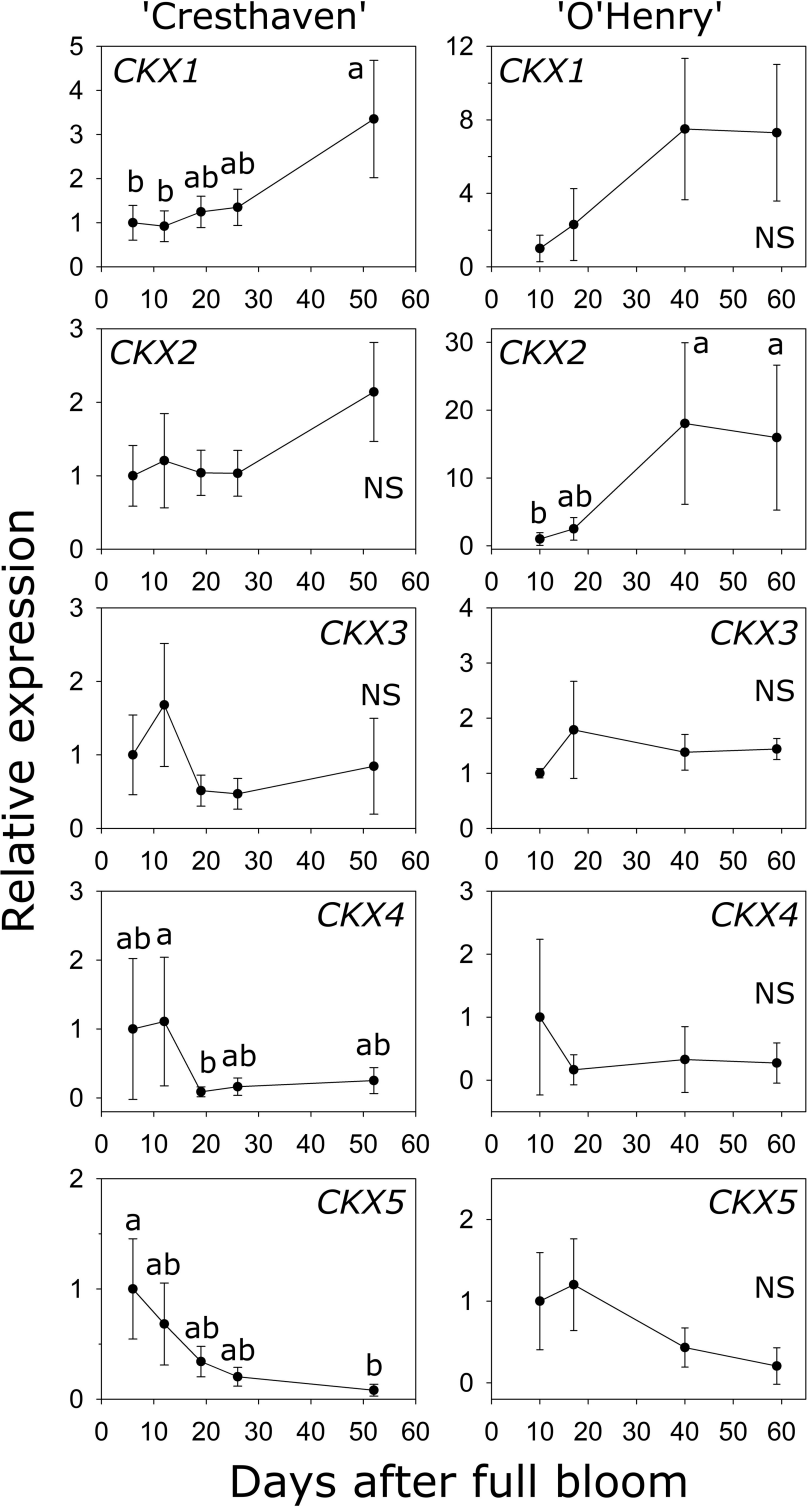
Transcript abundance of five *CYTOKININ OXIDASE* (*CKX)* genes potentially associated with cytokinin degradation in two peach genotypes. *β-ACTIN*, *KyACTIN*, and *RNA POLYMERASE II* were used as reference genes for ‘Cresthaven’ (*n* = 4). *β-ACTIN* and *RNA POLYMERASE II* were as reference genes in ‘O’Henry’ (*n* =3). Transcript abundance is presented relative to that at the first stage of fruit development. Fruit development stages were analyzed using ANOVA followed by Tukey’s HSD. Similar letters above the symbols indicate that the stages are not significantly different. NS: not significantly different across stages.

Five *CKX* genes were identified in peach (**Table S1**). *CKX1* abundance was higher by up to 3.6-fold between the early stages (6 and 12 DAFB) and 52 DAFB in ‘Cresthaven’ (**Figure 6**). Increase in *CKX1* transcript abundance in ‘O’Henry’ was not statistically significant. Conversely, increase in *CKX2* transcript abundance during fruit development was not significant in ‘Cresthaven’, but in ‘O’Henry’, it increased by over 18-fold between 10 DAFB and the last two stages evaluated (40 and 59 DAFB). *CKX3* transcript abundance was not significantly altered during fruit development in either cultivar. *CKX4* transcript abundance decreased during fruit development in ‘Cresthaven’ by over 13-fold between 12 and 19 DAFB and remained low thereafter but was unaltered in ‘O’Henry’. *CKX5* transcript abundance decreased gradually during fruit development in ‘Cresthaven’ by over 12-fold between 6 and 52 DAFB but was unaltered in ‘O’Henry’.

Transcript abundance of *CKX1* was negatively correlated with the concentration of *t*Z, *t*ZR and iP (*ρ* = −0.70) and with that of DHZ (*ρ* = −0.74). Further, transcript abundance of *CKX2* was negatively correlated with iP concentration (*ρ* = −0.70).

### Effects of fruit load reduction on fruit growth and cytokinin metabolism-related gene expression

Fruit thinning in ‘Cresthaven’ at 22 and at 29 DAFB increased fruit diameter by 6.9% and 9%, respectively (at first harvest), and fruit weight by 25% and 33%, respectively. Three of the five *IPT* genes (*IPT3*, *IPT5*, *IPT6*) displayed lower expression (by 2.1-, 1.8- and 2-fold, respectively) at 26 DAFB in response to thinning at 22 DAFB. Transcript abundance of *LOG4* and *LOG8* was significantly lower at 26 DAFB by 1.7- and 2-fold in the fruit thinning treatments performed at 29 DAFB compared to the control but were inconsistent with the timing of the imposed treatments. *LOG8* abundance was also lower than the control at 26 DAFB in fruit thinned at 22 DAFB (2.4-fold). *LOG6* transcript abundance at 52 DAFB was higher in fruit thinned at 22 DAFB than in control and 29 DAFB thinning treatments by 3.8- and 6.2-fold, respectively. Transcript abundance of *CKX* genes was generally unaffected by the thinning treatments, except for 3-fold higher abundance of *CKX4* at 26 DAFB in fruit thinned at 22 DAFB, compared to the control.

## Discussion

Early fruit growth in peach (until around 40 DAFB) was associated with intensive cell division within the pericarp. Peak RCPR noted during this period in ‘O’Henry’ coincided with the period of high RGR of the fruit and pericarp. Following this period, a sharp decline in fruit and pericarp RGR was observed and was associated with no further increase in cell number. Similarly, Scorza et al. (1991) reported a 11-fold increase in cell number between bloom and ~5 weeks after full bloom, a period they termed as Stage IA to reflect a period of active cell production during early fruit development. Cell number increase during this period was substantially greater than that noted at later stages (Stage II and Stage III). Hence, early fruit development until around 40 DAFB represents a period of intensive cell production in peach.

*Trans*-zeatin, iP and DHZ were the active cytokinin free bases quantified in this study. Quantification of *cis*-zeatin (*c*Z) was attempted but it could not be detected within the fruit tissue, suggesting that it does not constitute a major form of active cytokinin in peach. Among the active free bases, *t*Z was the major cytokinin during early fruit development and its abundance was highest during the early period following bloom and rapidly declined at later stages. Abundance of iP and DHZ increased during early fruit development and subsequently declined. These patterns of changes in the concentration of the cytokinin bases are similar to that noted in other fruits such as kiwifruit and tomato (Lewis et al., 1996; Matsuo et al., 2012). In these previous studies, high levels of active cytokinins around bloom and immediately thereafter have been associated with roles in fruit set. Similarly, high concentration of *t*Z and abundance of iP and DHZ during the earliest stages of fruit development may reflect roles for them in peach fruit set. Peak concentration of these active forms of cytokinins also coincided with periods of high cell production (peak RCPR) in the fruit pericarp, suggesting that they also play roles in regulating cell division during early fruit growth. Cytokinin activity is dependent on the ability to bind to cytokinin receptors, an essential feature for eliciting cytokinin responses (Spíchal et al., 2004; Romanov et al., 2006; Savalieva et al., 2018). *t*Z, iP and DHZ have all been demonstrated to bind to plant cytokinin receptors (Romanov et al., 2006; Lomin et al., 2015). Plants possess multiple classes of cytokinin receptors, and the binding affinities of cytokinins differ across these receptors, and across plant species (Spíchal et al., 2004; Romanov et al., 2006; Kieber and Schaller, 2014; Lomin et al., 2015; Lomin et al., 2018). As the binding affinities of peach fruit cytokinin receptors to *t*Z, iP and DHZ are currently unknown, the relative efficacy of the three bases in eliciting cytokinin responses cannot yet be deduced. It is possible that iP and DHZ may support cell production activity in the developing fruit, even at their relatively lower (3- to 4-fold) concentrations compared to that of *t*Z. Relative abundance of *t*ZR was around 8-fold higher than that of *t*Z during early fruit development. In a pattern parallel to that of the active forms, abundance of *t*ZR declined by several-fold during the early fruit development period. *t*ZR was previously reported to bind to cytokinin receptors in *E. coli* based assays, although with a relative affinity around 10-fold lower than that of *t*Z (Spíchal et al., 2004; Romanov et al., 2006). However, subsequent plant membrane-based assays clearly demonstrated that cytokinin ribosides display weak or undetectable binding to cytokinin receptors (Lomin et al., 2015). Hence, *t*ZR is not likely to function as a direct active cytokinin in peach fruit. Hence, *t*ZR may serve as an intermediate in the synthesis of *t*Z or as a transport form of cytokinin (Kieber and Schaller, 2014; Sakakibara, 2021).

Arnau et al. (1999) quantified various cytokinin metabolites within the developing peach fruit and identified the nucleotides, iPRMP and DHZRMP, and the nucleoside, DHZR, as the most abundant cytokinin metabolites, particularly during the early fruit development period. Specifically, these authors suggested that iPRMP abundance was correlated with cell division following fruit set in peach, as its abundance remained high during the cell production-associated early period of fruit growth. However, cytokinin nucleotides, although often abundant, are unlikely to display cytokinin activity, as bulky substitutions at the *N^9^* position of the adenine as those present in iPRMP, result in greatly decreased cytokinin activity (Savalieva et al., 2018; Romanov and Schmülling, 2022). Hence, iPRMP likely serves as an immediate precursor pool for conversion to active forms, for example *via* the direct activation pathway mediated by LOGs (Kurakawa et al., 2007; Kuroha et al., 2009). In the previous study in peach, *t*Z, iP and DHZ were identified as minor cytokinins based on their abundance but were reported to be specifically abundant in the pericarp at bloom (Arnau et al., 1999). Further, patterns of changes in their concentration in the pericarp during early fruit development were largely similar to those reported in the current study, particularly for *t*Z. Specifically, these metabolites were high during early fruit development and declined sharply by around 39 DAFB. These data further support the idea that these active cytokinin metabolites are closely associated with progression of cell production during early peach fruit development.

IPTs catalyze the rate limiting step of cytokinin nucleotide synthesis. Arabidopsis has seven IPT coding genes which display differential spatial expression patterns and sensitivity to cytokinin inducing or inhibiting factors (Miyawaki et al., 2004). For example, among the seven *IPTs*, *AtIPT7* expression is induced by KNOX1 (KNOTTED1-LIKE HEMEOBOX) proteins, resulting in an increase in cytokinin concentration in the shoot apical meristem (Yanai et al., 2005). In tomato, abundance of several *IPT* genes (*IPT1* and *IPT2*) increased during early fruit development, a period associated with active cell production (Matsuo et al., 2012). In the current study, transcript abundance of *IPT1*, *IPT2* and *IPT6* increased during fruit development but tended to remain high during later stages. Also, abundance of *IPT3* transcripts was high during early fruit development and declined later, but only in one of the two cultivars studied. Considering that several of the *IPT* genes were expressed in the pericarp tissue, and some such as *IPT2* at high abundance, it can be concluded that this tissue displays capacity for cytokinin biosynthesis during fruit development. This is also consistent with a previous report of the accumulation of cytokinin base precursors such as iPRMP during early peach fruit growth (Arnau et al., 1999). However, the lack of positive correlation of *IPT* transcript abundance with cytokinin concentrations and cell production, suggest either that *IPT* transcript abundance is not limiting for cytokinin biosynthesis in the developing fruit, or that post-translational regulation of IPT activity affects cytokinin biosynthesis during this period. In other plants, expression of *IPTs* and their specific localization to vascular tissues is associated with synthesis of transport forms of iP-type cytokinins (Miyawaki et al., 2004; Takei et al., 2004; Sakakibara, 2021). Lack of positive correlation between *IPT* expression with cytokinin concentrations in this study also suggests that iP-type cytokinins are synthesized and potentially transported out of the fruit pericarp to target sites such as the seed.

Transcript abundance of many of the *LOG* genes identified, was high during the earliest stages of fruit development, coincident with free cytokinin base abundance and with the period of cell production. Their abundance declined at later stages, coincident with declining cytokinin concentration and cell production rate. The transcript abundance of multiple *LOG* genes such as *LOG3*, *4*, *6* and *8*, was strongly correlated with the concentration of the free cytokinin bases, but not with *t*ZR. Among these, *LOG3* and *LOG8* displayed greater transcript abundance (based on their *NRQ* values) in the fruit pericarp during development. Interestingly, *LOG3* abundance was correlated with *t*Z concentration, while that of *LOG8* was strongly correlated with that of DHZ and iP, suggesting specific roles for their gene products in regulating the synthesis of free cytokinin bases. Such specificity may arise due to the specificity of LOGs for nucleoside monophosphate substrates. However, in Arabidopsis, LOGs did not display substrate specificity to different cytokinin nucleotides (Kuroha et al., 2009). Alternatively, the noted correlations and specificity may arise due to localization of LOGs and subsequent localized activation of cytokinins. In Arabidopsis, *LOGs* displayed differential spatial localization suggesting potential for localized activation of cytokinins (Kuroha et al., 2009). Further, specific localization of AtLOG4 to the L1 layer of the shoot apical meristem (SAM) leads to the generation of a cytokinin gradient which defines the expression domains of SAM regulators such as *WUSCHEL* (Chickarmane et al., 2012). Correlation between the expression of *LOG3* and *LOG8* and specific cytokinin bases may therefore reflect spatial localization of these LOGs within the pericarp. For example, specific *LOG8* expression in certain cell types of the fruit pericarp may lead to localized activation of iP and DHZ. Future experiments should aim to analyze such spatial localization of LOGs and cytokinin base abundance in greater detail. Together, these data indicate that the direct activation pathway is a potentially significant route for synthesis of active free bases during peach fruit development.

As *t*ZR was an abundant cytokinin compound in this study, part of the two-step cytokinin base synthesis pathway mediated by a nucleotidase and a nucleosidase may still be functional in the developing peach fruit pericarp. Alternatively, considering that *t*ZR is an important translocated form of cytokinin (Osugi et al., 2017; Sakakibara, 2021), *t*ZR abundance in the fruit pericarp may reflect its transport from a distinct source such as the developing seed. Arnau et al. (1999) reported that *t*ZR concentration in the seed increased during early stages of fruit development and suggested that this increase was associated with embryo development. *t*ZR can be converted to active cytokinin bases *via* their re-conversion to cytokinin nucleotides through the purine salvage pathway followed by conversion of the nucleotide *via* LOG to the active free base (Sakakibara, 2006; Tokunaga et al., 2012). Hence, it may be speculated that localized transport of *t*ZR from the seed to the developing pericarp tissue partially contributes to its abundance and subsequent active cytokinin formation in the fruit pericarp. Such transport of an active phytohormone precursor from the seed may help coordinate fruit development with that of seed development in peach (Bonghi et al., 2011).

Inactivation of active cytokinin bases is achieved either through the degradation pathway or *via* their conjugation (Kieber and Schaller, 2014). The CKX proteins irreversibly cleave the side chains from cytokinin bases such as iP an *t*Z but bases such as DHZ are relatively resistant to such cleavage (Kieber and Schaller, 2014). The CKX proteins may also use cytokinin ribosides, nucleotides and conjugated forms as their substrates (Galuszka et al., 2007). Over-expression of *CKX* in Arabidopsis resulted in reduced shoot growth (Werner et al., 2003). The gene underlying a QTL (Quantitative Trait Locus) associated with increased grain number production in rice was identified as coding for OsCKX2 (Ashikari et al., 2005). Reduced transcript abundance of *OsCKX2* results in increased cytokinin levels and higher grain number in rice. These studies demonstrate the significance of the degradation pathway in regulating cytokinin homeostasis. In the current study, transcript abundance of two *CKX* genes, *CKX1* and *CKX2*, was low during early fruit development and increased at later stages, in a pattern complimentary to that of changes in cytokinin concentrations. These data suggest that *CKX1* and *CKX2* play significant roles in the downregulation of cytokinin concentration during later stages of fruit development, following the period of peak cell production in the pericarp. However, differences across the two genotypes were evident with respect to the specific *CKX* gene associated with this role. Higher transcript abundance of *CKX5* during early compared to that at later stages is consistent with expression noted in other fruits such as tomato, and with previous reports indicating high abundance of *CKXs* in proliferating tissues (Werner et al., 2003; Matsuo et al., 2012). These data suggest a role for *CKX5* in fine-tuning tissue cytokinin concentration during early fruit development.

Crop (fruit) load reduction by manual thinning resulted in increased fruit growth and final size, consistent with previous studies (Njoroge and Reighard, 2008; Sutton et al., 2020). However, no consistent effects of thinning on the expression of cytokinin metabolism-related genes were noted in this study. It may be likely that the timing of fruit thinning (22 or 29 DAFB) was beyond the period where peak abundance of cytokinin concentration (active forms) occurred. Alternatively, fruit load reduction may influence peach fruit growth and cell division through mechanisms independent of changes in cytokinin metabolism.

Other phytohormones such as auxins and gibberellins are also thought to play important roles during early peach fruit development. Indole-3-acetic acid (IAA) concentration peaks during periods of rapid fruit growth suggesting a role in regulating the periods of rapid cell division and/or expansion in peach (Miller et al., 1987; Vizzoto et al., 1989; Masia et al., 1992). Similarly, GA_3_, an active form of GA, abundance is greatest during periods of rapid cell expansion in seed tissues and the mesocarp (Jackson, 1967). Cross-talk between IAA and GAs regulates fruit developmental processes such as fruit set in other fruits (Serrani et al., 2007; Serrani et al., 2008). In peach, changes in transcript abundance of phytohormone-responsive genes in the fruit mesocarp suggests that such potential interactions occur in the developing peach fruit (Bonghi et al., 2011). Hence, it may be proposed that cross-talk among these phytohormones and cytokinins likely regulates the extent of cell production, and developmental transitions from cell division to cell expansion during fruit growth in peach. Such interactions warrant further investigation.

## Conclusion

The abundance patterns of active cytokinins during early stages of fruit development are consistent with a role for these phytohormones in regulating the cell division–mediated phase of early fruit development in peach. Relatively high abundance of several *IPTs* in the fruit pericarp also indicate that these tissues can synthesize cytokinins. Multiple genes in the *LOG* family displayed expression patterns consistent with changes in active cytokinin free base abundance. Hence, active cytokinin forms are likely generated in the fruit pericarp *via* the direct activation pathway during early peach fruit development. Cytokinin catabolism during later stages of early fruit development may be partially mediated by *CKX1* and *CKX2*, resulting in the exit of cells from the cell production phase. Together, several cytokinin-metabolism-related gene products identified here may help fine-tune cytokinin homeostasis during early fruit development in peach.

## Data availability statement

The original contributions presented in the study are included in the article/**Supplementary Files**. Further inquiries can be directed to the corresponding author.

## Author contributions

MS, DC, and AM conceived the study, and designed and executed the experiments. MS and BR collected data and processed samples for gene expression analyses. BR and AM collected and processed samples for cytokinin quantification and microscopy analyses. MS, BR, and AM were involved in data analyses. MS and AM prepared the draft of the manuscript. All authors contributed to the article and approved the submitted version.
