# Supplementary material for: Synthesis of active cytokinins mediated by LONELY GUY is associated with cell production during early fruit growth in peach [Prunus persica (L.) Batsch]
Source: Front Plant Sci. 2023 Apr 20;14:1155755. doi: 10.3389/fpls.2023.1155755 (PMC10157650; doi:10.3389/fpls.2023.1155755)
Supplement: Supplementary file 1 [file DataSheet_1.pdf]

Synthesis of Active Cytokinins Mediated by LONELY GUY (LOG) is Associated with Cell Production During Early Fruit Growth in Peach [*Prunus persica* (L.) Batsch]

Mary Sutton[1], Bayleigh Roussel[1], Dario Chavez[2] and Anish Malladi[1]

[1]Department of Horticulture, University of Georgia, Athens, Georgia, USA 30602

[2]Department of Horticulture, University of Georgia, Griffin, Georgia, USA 30223

Supplementary Data

Table S1. List of *IPT*, *LOG* and *CKX* genes identified in peach and sequences of primers used for quantitative RT-PCR (5´ – 3´).

Gene	Gene Identity	Forward	Reverse
IPT1	Prupe.1G150800.1	CGGTGGCTCGAATTCCTACA	GCCTTCCTGATTCCGTGAGT
IPT2	Prupe.1G151100.1	GCAAGTCTACAGAGGCCTGG	CCACCGTCCGATCGATGTAG
IPT3	Prupe.4G170400.1	TTGATAGTGGCCGTGTTCCC	CAGCTGCTTCCCAGTCTTCA
IPT5	Prupe.6G12800.1	GATGCTTCTCTCCCTGTGCT	ATCAGGCAGGTCTACTGACG
IPT6	Prupe.8G243600.1	TCCATTTTAGGCCGGGATCG	TGTCCACCCGTTTTGACACA
CKX1	Prupe.1G373300.1	CTGTTCTTGGTGGGCTAGGG	AACTTGTGGGTGGAGGGTTG
CKX2	Prupe.1G404300.1	GGACGAGGAGGACTCGTTTG	AGTAGAGCATTGATCCGGCG
CKX3	Prupe.2G026700.1	CGGGCGAGAATATCGTTGGA	CCTGAACCGGGTCCTTTGAA
CKX4	Prupe.7G052300.1	TTCGCCTATGTACGGCTTCC	TGTTGGTGTGGCAAGGAGTT
CKX5	Prupe.7G208400.1	AACATTACCACCGCACCAGT	CAAGTGCCTCCCACTCATCA

LOG1	Prupe.1G367500.1	CTGGGAAGAACCC GAGCTAC	AGACAGCTTGGGA GACCTGA
LOG2	Prupe.1G409400.1	GACATGCATGAGC GTAAGGC	CCGAGCACATGGC TTGATGA
LOG3	Prupe.4G230500.1	GGGTCAGAGCTTC CAGTTCC	TGCTGGGCTTTGTC AGTAGG
LOG4	Prupe.6G005100.1	GGGACTCAACTGG TGGAGAG	TCAAGTGGAACAA GGGCTGT
LOG6	Prupe.6G236000.1	TAAACCGGTGGGA TTGCTGA	GTGTAGCCAAGCT GCTCCAT
LOG7	Prupe.7G097700.1	CCTCTTGAATGTG GACGGCT	GCCAACACATCAC GTGGTTC
LOG8	Prupe.8G174600.1	GGGCACAGCTTGG TATCCAT	GCTCAACCTCCCA TCTTGCT

Table S2. List of reference genes and the primer sequences used for quantitative RT-PCR (5´ – 3´).

Gene	Forward	Reverse
β-ACTIN[a]	GTTATTCTTCATCGGCGTCTTCG	CTTCACCATTCCAGTTCCATTGTC
Ky-ACTIN[b]	GATTCTGGTGATGGTGTGAGT	GACAATTTCCCGTTCAGCAGT
RNA POLYMERASE II[c]	TGAAGCATACACCTATGATGATGAAG	CTTTGACAGCACCAGTAGATTCC

[a]Wang et al., (2016); [b]Haider et al., 2018 ; [c]Zhang et al., 2016

References cited

Haider, M.S., Kurjogi, M.M., Khalil-ur-Rehman, M., Pervez, T., Songtao, J., Fiaz, M., Jogaiah, S., Wang, C., Fang, J., 2018. Drought stress revealed physiological, biochemical and gene-expressional variations in ‘Yoshihime’ peach (*Prunus Persica* L.) cultivar. J. Plant Interac. 13, 83–90.

Wang, D., Gao, Z., Du, P., Xiao, W., Tan, Q., Chen, X., Li, L., Gao, D., 2016. Expression of ABA metabolism-related genes suggests similarities and differences between seed dormancy and bud dormancy of peach (*Prunus persica*). Front. Plant Sci. 6, 1248.

Zhang, C.H., Zhang, B.B., Yu, M.L., Ma, R.J., Song, Z.Z., Korir, N.K., 2016. Isolation, cloning, and expression of five genes related to nitrogen metabolism in peach (*Prunus persica* L. Batsch). J. Hort. Sci. Biotechnol. 91, 448–455.
